# Designing for downsizing: Home-based barriers and facilitators to reduce portion sizes for children

**DOI:** 10.3389/fpsyg.2022.915228

**Published:** 2022-10-03

**Authors:** Tang Tang, Wenmeng Wang, Marjan Vazirian, Fiona Croden, Marion M. Hetherington

**Affiliations:** ^1^School of Design, University of Leeds, Leeds, United Kingdom; ^2^School of Art and Design, Wuhan Polytechnic University, Wuhan, China; ^3^School of Psychology, University of Leeds, Leeds, United Kingdom

**Keywords:** packaging design, children, food intake, portion size, downsizing, portion control, home based studies

## Abstract

Evidence confirms that parents know that they should limit non-core foods for their children since these tend to be high in energy density (HED), fat, salt and sugar. However, it is unclear how knowledge of portion size limits, such as the 100 kcal guide from Public Health England are applied in practice. To observe in real-time children’s home food environment related to portion control and to explore with parents their reported portion size strategies, a mixed methods study was designed. Families with children aged 1–5 years were recruited (*n* = 21) to a three-part study: (1) to complete questionnaires and interviews on household food intake and portion control; (2) to report daily food intake for 4 days (*n* = 13) for one parent and their child(ren); (3) to observe home-based food provisioning *via* videorecording during dinner, breakfast and snack time (*n* = 6). Although the problem of large portion sizes of HED foods was recognised by mothers, strategies to downsize portions were not necessarily applied at home, as revealed in home observations and diaries. A mismatch between what was observed at home, what was reported in food diaries and what was said in interviews became apparent for some families. Mothers reported the need for greater support and guidance to downsize HED foods since they relied on pre-packaging as a guide to intake. Education and engagement were identified as important parameters for downsizing by mothers. One strategy which could be explored and applied by manufacturers is packaging design to faciliate the 100 kcal guidance using physical and engaging ways to assist parents in downsizing HED foods for their children. To facilitate effective government communication, innovative packaging design can be used to convey clear guidance and to tailor portion size messages for children. Packaging design, alongside government recommendations, can support parents’ goals to achieve healthy eating and can reinforce guidance for portion norms through innovation involving learning, playful engagement, and interaction.

## Introduction

The portion size effect (PSE) is a phenomenon in which large portions of foods offered to participants encourages large intakes, it has been demonstrated in several laboratory studies (Rolls, 2003; Rolls et al., 2004; Pourshahidi et al., 2014; Zuraikat et al., 2019) and is robust and reliable in young children (Birch et al., 2015; Smethers et al., 2019) including in the context of vegetable consumption (Roe et al., 2022). Offering large portions of high-energy-dense (HED) can lead to overconsumption in children (Zlatevska et al., 2014; Kral and Hetherington, 2015) and may contribute to the increasing prevalence of childhood overweight and obesity (Scottish Maternal and Infant Nutrition Survey, 2018). HED foods are defined as those with an energy density > 4 kcal/g (see British Nutrition Foundation)^1^. The World Health Organisation has proposed that limiting portion sizes to reduce overall energy intake could prevent unhealthy weight gain [World Health Organisation (WHO), 2014]. In 2018, the UK Government through Public Health England introduced guidance to parents to offer children no more than two snacks per day, each of 100 kcal or less. This guidance prompted food and beverage companies in the UK to reduce the portion size of packaged snacks to facilitate portion control for children, although it remains the case that most packaged HED items provide suggested serving sizes for adults, not children. Snack package sizes are, on average, about 2.5 times larger than appropriate for young children (Sothern, 2004).

The Infant and Toddler Forum 2015 survey found that UK children aged 2 years were offered adult portions of HED snacks. This means that children may be consuming more fat, sugar and salt than is recommended. Sugar intake accounted for 13.5% of 4 to 10-year-olds daily energy intake, which is above the 10% of total energy intake suggested by the World Health Organisation (WHO) (2015), and half of the children’s sugar intake was derived from HED snacks (Public Health England, 2018). In addition to consuming more energy from sugar than recommended, in receiving adult size portions of some foods, children may expect to receive these as the norm (Robinson et al., 2016). In summary, children are susceptible to the PSE, are often given (and expect) adult size portions of HED foods and are liable to making perceptual errors in judging portion size (Frobisher and Maxwell, 2003), particularly when adult portions are presented to them (Foster et al., 2006). Thus, there is a need to both offer downsized servings of HED items for children and guidance to promote consumption of age-appropriate portion sizes in young children in the home environment (Porter et al., 2021).

If children are offered and are eating adult size portions of some HED foods, this may contribute to overconsumption and over time, to overweight and obesity. In the latest results from the UK government’s National Child Measurement Programme (2021) the proportion of children in Year 6 (aged 10–11 years) living with obesity was almost double (25.5%) that in Reception (aged 4–5 years; 14.4%). Early diet and eating patterns in childhood track into later life (Nicklaus et al., 2005), highlighting the importance of establishing age-appropriate consumption norms in the early years. For children, the home food environment is pivotal in predicting, shaping and controlling exposure to large portion sizes of palatable, energy-dense foods (Hetherington and Blundell-Birtill, 2018) and mothers are most likely to be the gatekeepers of this home food environment. Therefore, the current study was designed to explore the home food environment, food provision by mothers and barriers/facilitators to portion control especially for prepackaged foods.

Food packaging is only one of many environmental cues which can influence food consumption decisions (Antonuk and Block, 2006) and intake (Ares and Deliza, 2010; Keller et al., 2012), as well as being used to promote HED foods to parents and children (Kiefner-Burmeister and Burmeister, 2021). Parents make portion size decisions for their children based on a range of information sources including package sizes (Blake et al., 2015) and labelling. A study of nutrition-related claims under current labelling practices led parents to choose less healthy drinks for their children and misled them about the healthfulness of fruit drinks, which may guide choice and consumption (Harris and Pomeranz, 2021; Hall et al., 2022).

A systematic review of packaging manipulation found that packaging features can be used to downsize the intake of HED foods and increase consumption of nutrient-dense foods (Chu et al., 2021). In particular, large pack sizes (Aerts and Smits, 2017), large suggested portion sizes of food on the front of the packaging (Neyens et al., 2015; McGale et al., 2020) and recognisable brand logos (Keller et al., 2012) act to facilitate food intake in children. Parents report that packaging is an important determinant of food product purchase decisions and can offer a useful, convenient means of portion control (Tang et al., 2020).

This study was designed to observe in the home environment, the extent to which mothers use and need packaging to aid portion control of highly liked HED foods for their children using a mixed-methods approach (qualitative and quantitative). The specific objectives of the study were: (1) to understand real-world use of food packaging involving children through unobtrusive videorecorded observations during meal and snack preparation and intake at home; (2) to characterise typical food intake, including portions offered to children of HED foods, through food diaries; (3) to explore both observed and reported needs for packaging to manage portion sizes of snacks and meal items through observations and interviews. Triangulation between direct observations, interviews, and food diaries, provides a robust platform from which to optimise understanding of everyday aids to support healthy behaviours in children (Won and Tang, 2017). We hypothesized that portion control could be achieved through food packaging design as well as the implementation of portion control aids, as previously reported in the literature. However, it is not known to what extent parents use packaging as a solution to support appropriate portion size for their children. The following research questions were addressed through a range of home-based studies: (1) whether packaging was used at home for portion control and how it was used; (2) what kind of HED snacks the children were eating and what proportion of total daily energy intake was derived from snacks; (3) how packaging design can support appropriate portion size for children.

## Materials and methods

A mixed-methods approach was applied to gather data in the home environment. Qualitative methods included video-recorded home observations of food preparation and food intake at breakfast, snack time and dinner, and semi-structured interviews. Quantitative methods included self-reported questionnaire and food diary data. The study was given ethical approval *via* the Faculty of Performance, Visual Arts and Communications (PVAC) Ethics committee (ref PVAR 15–096), the University of Leeds, United Kingdom.

### Participants

To minimise the demand characteristics of the study (Verplanken and Faes, 1999), participants were invited to participate in an ‘eating habits’ study. Families were recruited from Leeds, Manchester and Chester through convenience sampling. Due to the requirement of a four-day consecutive food diary (including at least one weekend day), parents were excluded if their child attended nursery for more than three full consecutive days. Parents were also excluded if their child had breakfast and/or evening meals outside the home. Inclusion criteria included having primary responsibility for feeding their child most of the time and being the biological parent of a child without a chronic medical condition affecting growth or eating (e.g., food allergies or intolerances, developmental disorders, or birth defects). All eligible participants identified from the screening questionnaire were invited to participate in all three parts of the study, including home-based observation, food diaries and semi-structured interviews, as shown in Table 1. In 12 months ending Feb 2018, in total, 21 mothers aged between 24 and 51 years with 25 children aged 1–5 years (range 16–57 months) participated in the study. Mothers could select which tasks they were able to do. All mothers agreed to the questionnaire and interview, 13 mothers agreed to do the four-day, weighed food diaries, and six agreed to the video observation.

**Table 1:** Overview of research methods used, duration of each task and sample size.

Research methods	Duration	Group 1 (*n* = 6)	Group 2 (*n* = 7)	Group3 (*n* = 8)	Total
Family profile questionnaire	10 min	x	x	x	21
Observational study (video camera situated at home)		x			6
One evening meal and breakfast	60 min				
One snack time	15 min				
Weighed and photographic food diary	4-day	x	x		13
Project debrief. Semi-structured interview and Comprehensive Feeding Practices Questionnaire (CFPQ)	90–120 min	x	x	x	21

### Procedure

After consenting to the study, mothers completed a short family profile questionnaire (typically used in our laboratory to collate general demographics, Supplementary Material A) to help characterise the household. Then those mothers who had agreed to home observation and diet records (Groups 1 and 2) were visited at home to familiarise them with the research protocol and with the researchers. During this visit, instructions for both video observation and food diaries were provided.

To aid unobtrusive video recording, two compact size cameras with a motion sensor (Conbrov® DV9 HD Book Camera) were set up in participants’ kitchen and dining room for the household observation. This usually started between 5 and 6 pm with the recording until 9 and 10 am following day, depending on usual eating times. The cameras were placed strategically to get the best view to capture what and how the child(ren) ate for breakfast and dinner, then a Go-Pro camera was used to film a snack time. As the cameras were small, there were easily positioned and inconspicuous.

Mothers in Group 2 who chose to participate in the diary study were instructed to keep a weighed food and drink diary for their child and themselves, for four consecutive days (Thompson and Subar, 2017).

At the end of the study, all parents were interviewed using a semi-structured interview guide and then they were fully debriefed about the study’s objectives. The interview and debriefing visit took place after the completion of all home observation and diary data collection (Table 1). Participants in groups 1, 2 and 3 were compensated for their participation with £30, £20 and £10 supermarket gift vouchers, respectively. An additional £10 gift voucher was given to complete the food diary for a second child in the studied age group.

### Materials

The short family profile questionnaire (as mentioned above) was used to collect demographic data in person. Information regarding child care (day per week), food shopping patterns and child eating habits was collated to ensure that participants met inclusion criteria and to prepare for the observational studies. For diet records, detailed instructions, photographic examples, the weighing scales (Salter Aquatronic Digital Kitchen Scales) and a demonstration on how to use the scales accurately were all provided. All foods and beverages consumed by the mothers and their child inside and outside of the home were included, without changing their habitual diet. Mothers were also asked to take photos of foods and beverages served before and after eating to provide additional data on portion sizes offered. They were also asked to bring in packages of food items, from which more accurate nutritional analyses could be conducted. The interview was conducted with a semi-structured guide lasting between 60 and 90 min (Supplementary Material A), with six sections covering parents’ perceptions of portion size, the child–parent purchase relationship, feeding practices and portion size strategies, intention and confidence (self-efficacy beliefs) to serve age-appropriate portions of meals and snacks, and parents’ perceptions and needs of packaging solutions for downsizing. The Comprehensive Feeding Practices Questionnaire (CFPQ; Musher-Eizenman and Holub, 2007) was administered to mothers to provide an overview of parental feeding practices. The CFPQ is a validated, self-report instrument composed of 49 items distributed over 12 factors to measure child-feeding practices, and each item is scored using a 5-point Likert scale.

### Analyses

The family’s sociodemographic characteristics (i.e., age, sex, and race/ethnicity) were summarized using descriptive statistics, including frequency (percentage) for categorical variables. Snacks were defined as foods or drinks eaten between main meals (see Chaplin and Smith, 2011). Each food/beverage item was entered from the diaries into an excel sheet by a trained research dietician (FC) then calculated weights and energy (kcal) intake from the weights given in the diary for each item using nutritional composition software WinDiets®. The WinDiets® software comprises two food databases, namely UK Food Tables 2017 and USA Food Tables 2017. One-sample t-tests were carried out using SPSS (IBM SPSS Statistics v20, Armonk, NY, United States) to examine differences in the average energy intake per day between the diary from our participants and the British Nutrition Foundation (2016) energy requirements in the UK. The mean (± standard deviations) for the weight (g) and energy intake (kcal) for snacks and total daily food intakes were calculated. This was done to compare amounts eaten by children to recommended portion sizes (where given). Outcome measures were: mean daily energy intake (kcal) of the parents and children, percentages of energy intake derived from savoury snacks and sweet snacks (including sweets, chocolates, cakes/biscuits and ice cream with an energy density > 4 kcal/g) and mean frequency of snacks eaten by the child and parent participants. Mean intakes were calculated from fruit, vegetables, snacks, drinks and total (g; snacks, meals and beverages combined). One-sample t-tests were performed to examine differences in CFPQ parental feeding practices against published norms. Alpha was set at *p* < 0.05.

Video recordings of breakfast, dinner and snack time were watched and described along with photographic food diaries to capture and reflect the use of packaging to determine the portion sizes. Attention was paid to occasions where packaging was or was not used by parents and children as a portion control measure. All interviews were recorded, transcribed, and then analysed. The transcripts from the interviews and description of the video recordings were analysed using thematic analysis (Braun and Clarke, 2006). Internal validity was adhered to by the process of comparison during the data analysis (by TT and WW). Discrepancies were discussed with MH until consensus was achieved (Ryan and Bernard, 2000).

## Results

### Demographics and questionnaire results

A total of 21 mothers (24–51 years., mean age: 35.1 years) with 25 children aged 1–5 years (Table 2; Supplementary Material B) completed the study over 12 months, in 2018 before the pandemic. Group 1 (*n* = 6, 24–39 years, mean age: 33.0 years) participated in all activities. Group 2 (*n* = 7, 30–51 years, mean age: 38 years) completed the questionnaires and food diaries (one for the adult participant and one or two for the child participants within the studied age group) and were interviewed. Group 3 (*n* = 8, 31–39 years, mean age: 34.5 years) were interviewed and completed the questionnaires only.

**Table 2:** Participant characteristics.

Participant characteristics									
	Attribute	All	Group1		Group2		Group3	
		Tot	%	Tot	%	Tot	%	Tot	%
**Mother**					MU01-MU06		MUd01-MUd07		MUi01-MUi06
Age (years)	21–30	3	14%	2	33%	1	14%	8	100%
	31–40	16	76%	4	67%	4	58%		
	41–50	1	5%			1	14%		
	51–60	1	5%			1	14%		
Age range			24–51		24–39		30–51		31–39
Mean age			35.1		33%		37.9		34.5
BMI (kg/m^2^)^**^	Underweight	2	10%			1	14%	1	12.5%
	Normal weight	15	71%	5	83%	4	57%	6	75%
	Overweight	4	19%	1	17%	2	29%	1	12.5%
Ethnicity	White British	11	52%	4	66%	4	57%	3	38%
	African	1	5%					1	12%
	Other White/white Irish	1	5%	1	17%				
	Chinese	8	38%	1	17%	3	43%	4	50%
Highest education	Vocational Qualification (GNVQ or BTEC)	2	10%	1	17%			1	13%
	Higher national certificate or Diploma	5	24%			3	43%	2	26%
	Undergraduate degree	6	28%	2	33%	1	14%	3	35%
	Postgraduate qualification (master/ PhD)	8	38%	3	50%	3	43%	2	26%
Employment status	Full time	5	24%	2	33%			3	38%
	Part-time	8	38%	2	33%	3	43%	3	38%
	Not working	8	38%	2	33%	4	57%	2	24%
Marital status	Two-parent/caregiver family	20	95%	6	100%	7	100%	7	88%
	Single parent	1	5%					1	12%
Income	£10–20,000	4	19%	1	16%	2	28.5%	1	13%
	£20–30,000	4	19%	1	16%	1	14%	2	26%
	£30–40,000	7	33%	2	34%	2	28.5%	3	35%
	£40,000+	6	29%	2	34%	2	28.5%	2	26%
**Child**									
Sex	Female	8	32%	1	13%	2	25	5	56%
	Male	17	68%	7	87%	6	75	4	44%
Age (months)	12–24	4	16%	2	25%	1	12	1	11%
	25–36	9	36%	1	12.5%	4	50	4	45%
	37–48	2	8%	1	12.5%			1	11%
	49–60	10	40%	4	50v	3	38	3	33%
BMI centile^*^	Overweight	5	20%	2	25%	2	25	1	11%
	Normal weight	18	78%	6	75%	5	62	7	78%
	Underweight	2	8%			1	13	1	11%

* Body mass index (BMI) centile based on Boys & Girls UK-WHO Growth Chart 0-4 years, Boys & Girls UK body mass index 2–20 and child’s height and weight reported by parents. Overweight is defined as > 91st centile and underweight < 2nd centile.

** BMI calculated from self-reported height and weight and classified as underweight <18.5 kg/m^2^; normal weight 18.5–24.9 kg/m^2^; overweight 25–29.9 kg/m^2^; obese >30 kg/m^2^.

For reports of interview content, quotations are labelled as follows: mothers are coded as MU, and then this code is linked to a numbered child ID with sex indicated (daughter = D, son = S) and child age. Most mothers identified as White (*n* = 12, 57%) and 38% as Chinese. Most families consisted of two-parent or caregiver families (95%) and most reported having a healthy weight (71%: self-reported). Approximately 90% of mothers had a higher national certificate or diploma. Two-thirds had an undergraduate degree or postgraduate qualification (Masters/PhD), with 62% of households earning above the average income for 2018 in the UK (ONS, 2018). Therefore, the sample was skewed towards more affluent and educated than the average UK household. Most children were male (68%) and had a healthy weight (78%).

In comparison to published norms for the Comprehensive Feeding Practices Questionnaire (CFPQ) by Musher-Eizenman and Holub (2007) study (Supplementary Material C), mothers in this study reported greater use of Restriction for Health – parental control of child’s intake to limit unhealthy foods; Pressure – parents’ encouragement of the child to eat more food at meals, disregarding child’s satiety or hunger, Food as Reward - parents’ use of food as a reward for child’s behaviour; Behaviour and Emotion Regulation - parents’ use of food to regulate the child’s emotions. However, Cronbach’s alpha for this small sample size was variable, and so these results must be treated with caution.

### Home-based observations

#### Parents’ use of situational cues

Video recordings showed that parents were using environmental cues including to determine how much to serve their child at home, including: (1) dishware; (2) pre-packaged individual foods; (3) reusable children’s food and drink packaging and (4) visual representation of the portion on the packaging.

**Figure 1:**
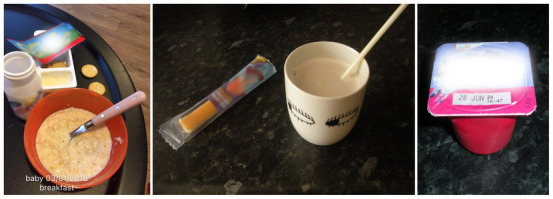
MUd05 (left panel) and MU01 (middle and right) using child-sized bowls, cups and pre-packaged snacks for children (MUd05D:25 m; MU01S1:48 m).

**Figure 2:**
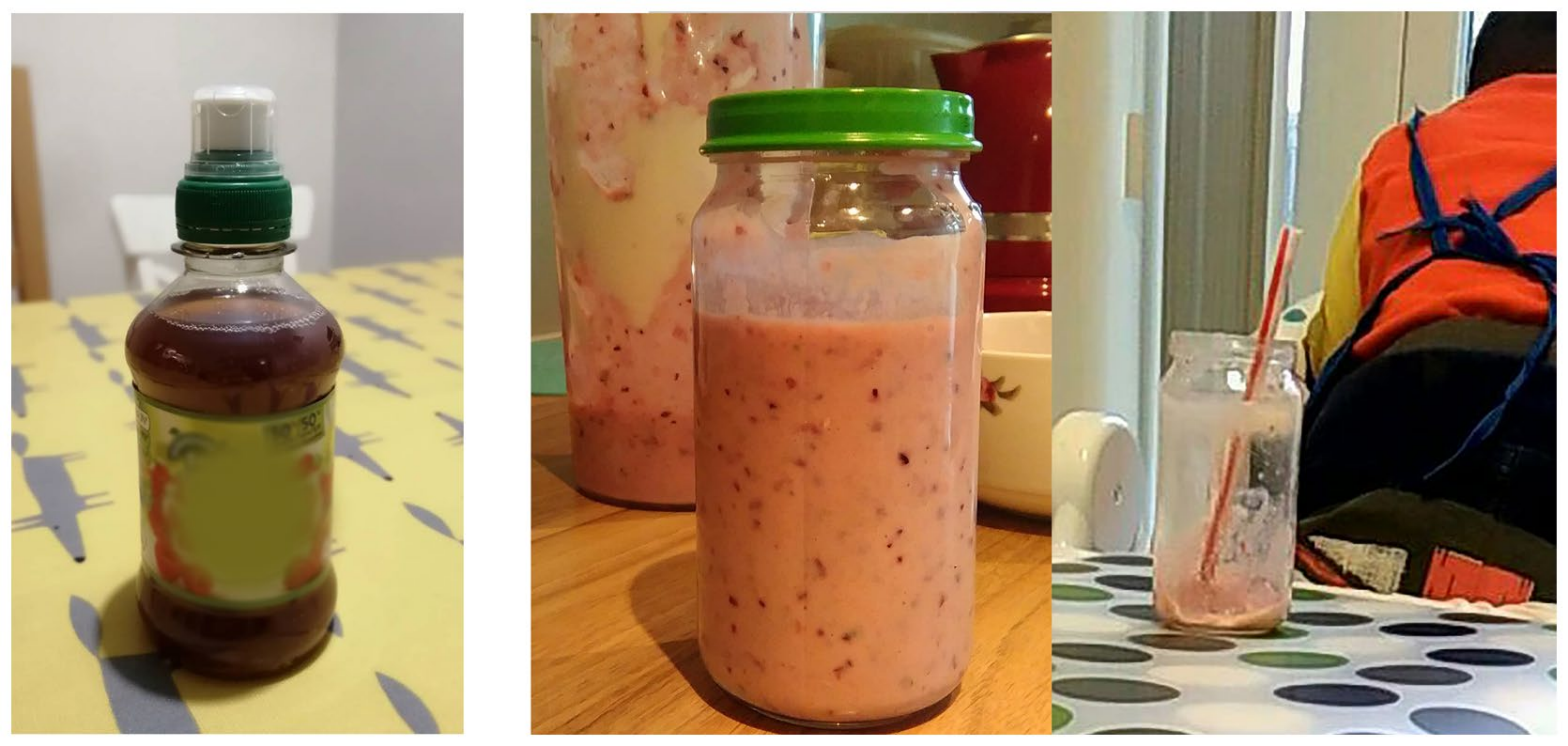
Reuse of children’s food packaging.

**Figure 3:**
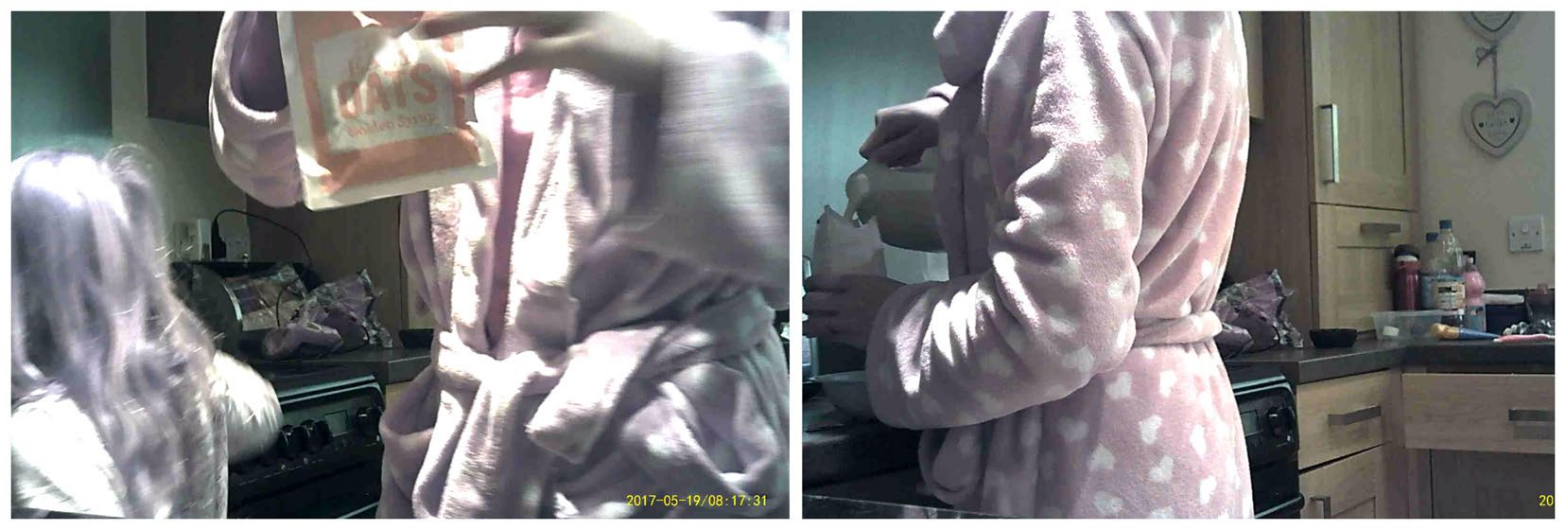
Subdividing large portions according to the visual representation of the portion on the packaging.

1. *Use of dishware*: dishware assisted portion control for children *via* smaller than adult sized/standard bowl and cups sizes (see Figure 1).
2. *Pre-packaged individual foods*: mothers often relied on the size of pre-packaged foods as a guide to appropriate portion sizes. Figure 1 shows that MUd05 and MU01 served breakfast items with individual serving portions to their children.
3. *Reuse of children’s food packaging*: children’s disposable food and drink packaging was reused as a reference portion size for their children. MUd05 (Figure 2 left panel) reused a children’s juice bottle for her daughter (MUd05D:25 m) outside the home. The snack observation showed that MU03 reused a Heinz 7-month baby meal jar to determine the amount of homemade fruit yoghurt offered to her three years old MU03S:36 m (Figure 2 right panel).
4. *Visual representation of the portion on the packaging*: large portions were subdivided according to visual representation on the packaging. MU05 poured one sachet of breakfast oats into a bowl and then used the sachet to measure the milk, filling it to the milk line. The milk line on the sachet is a visual aid depicting the appropriate amount of milk needed to make the porridge (Figure 3).

#### Packaging used by children

Video observations revealed that children helped themselves to food packaging, offered help to younger siblings by opening these for them and interaction with parents, *via* packaging.

**Figure 4:**
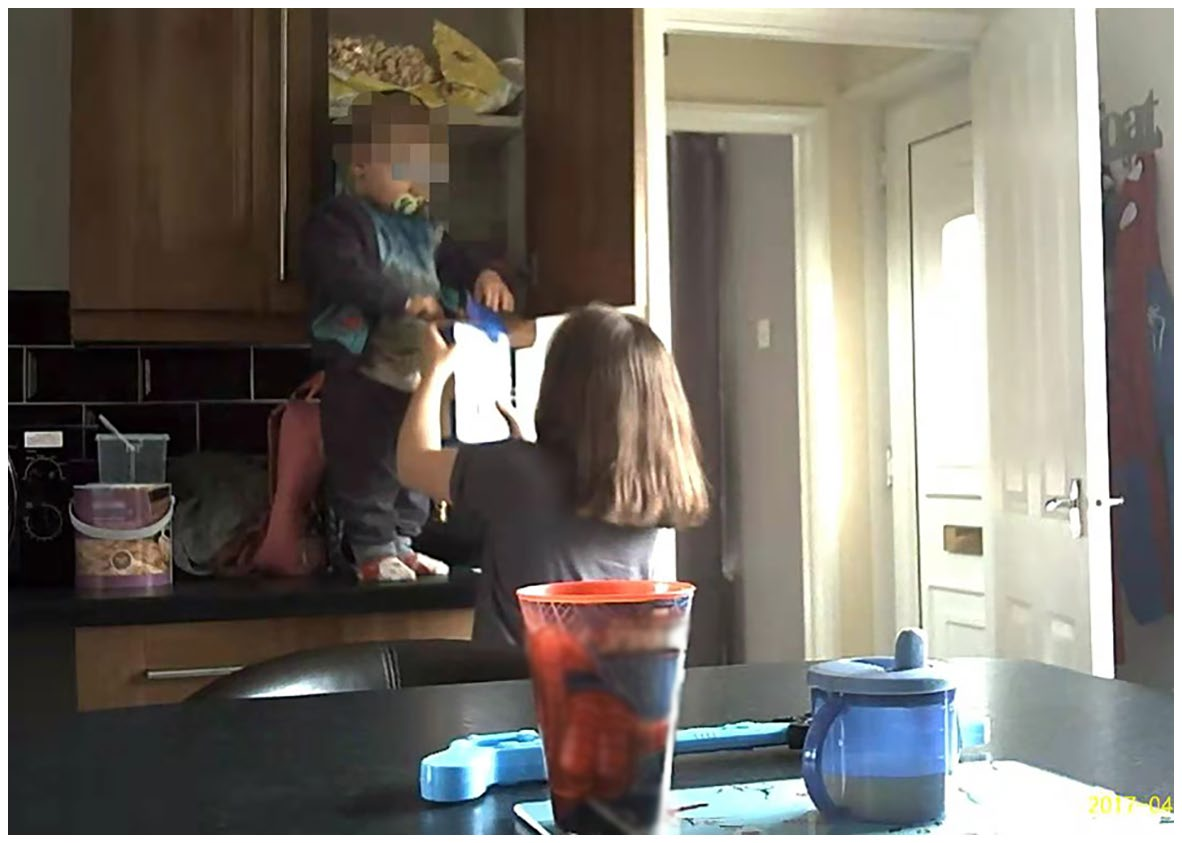
Packaging used by children (MU04).

**Figure 5:**
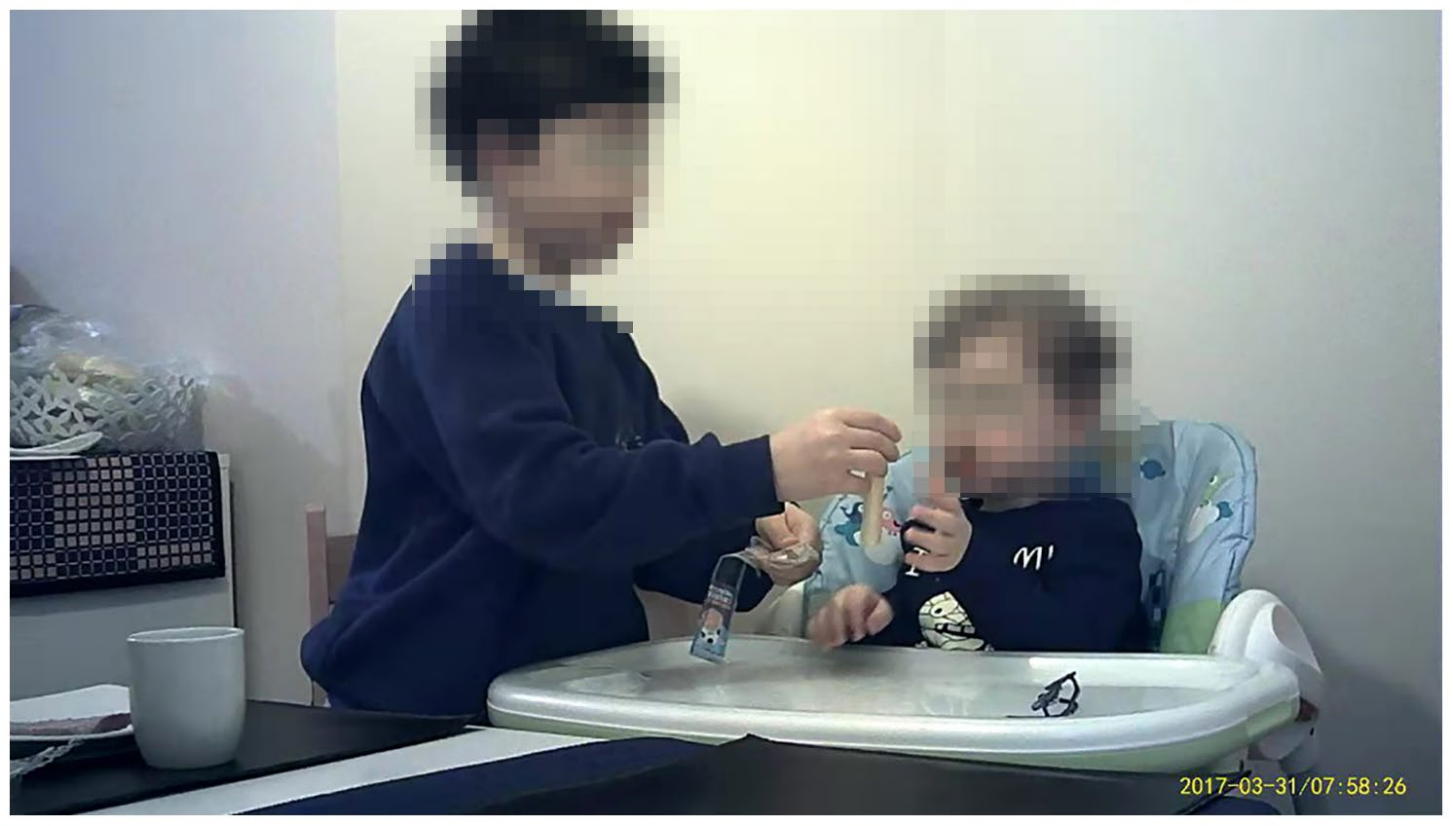
Helping others (MU01).

**Figure 6:**
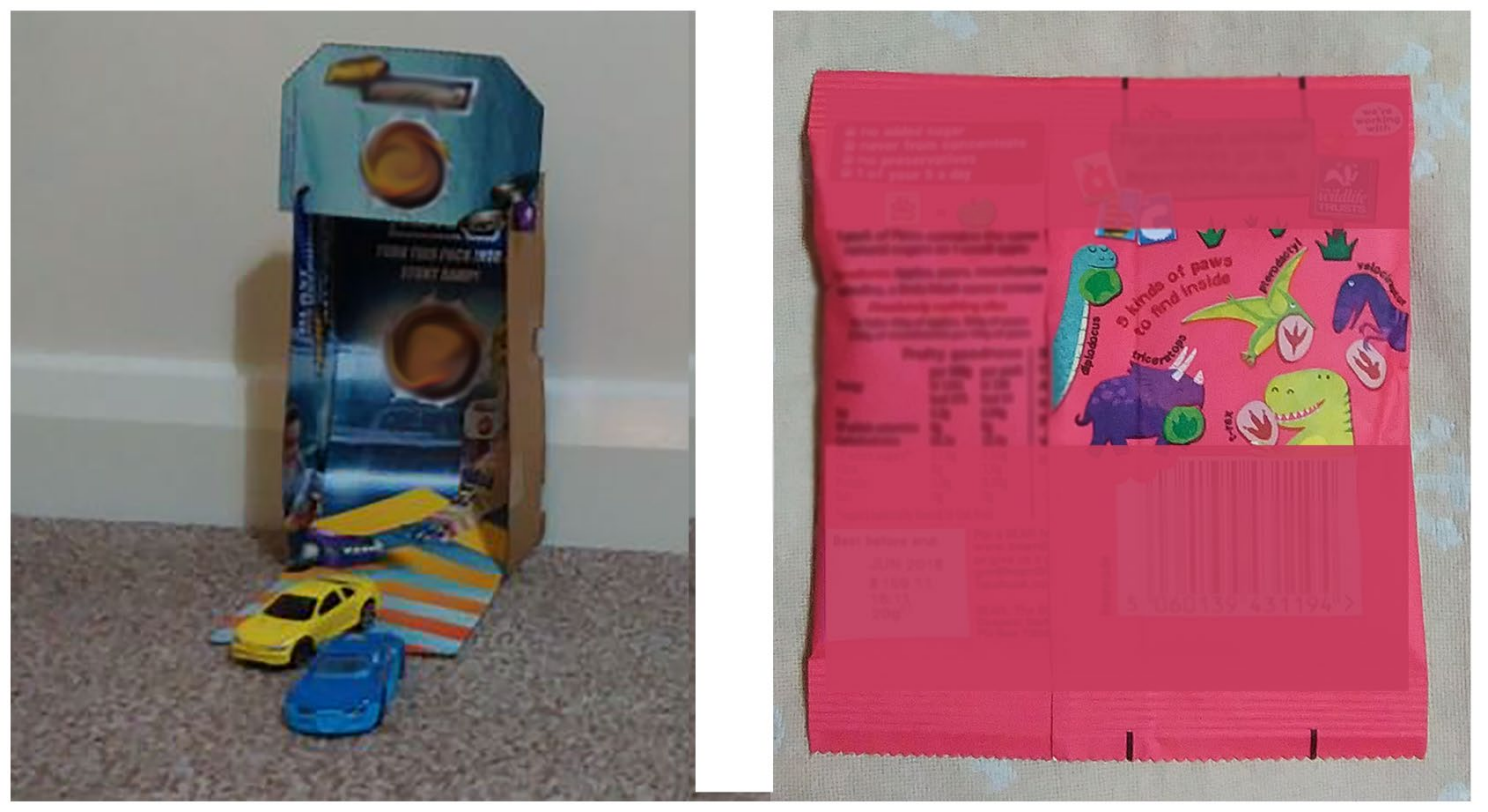
Packaging-led interaction.

1. *Self service*: food packages are identifiable, memorable and attractive to children. In the case of one household with three children, at 7:04 am, the 19-month old (MU04S1:19 m) climbed up to the cupboard and fetched the cereal he wanted for breakfast (Figure 4).
2. *Helping others*: some older siblings helped younger siblings by opening food packaging, particularly when parents were immersed in school morning routines (e.g., MU04; MU01). For example, at 7:58 am Friday, MU01S1:48 m started his breakfast with a cheese string, but could not open this by himself. MU01S2*: 8y9m his elder brother came to help and opened the packaging for MU01S1 (Figure 5).
3. *Increased interaction*: food packaging has the potential to encourage interaction between children and parents. MU03 made the car ramp with her three-year-old using a cereal packet and showed how she used a dry fruit package with a small interactive game (Figure 6) to slow down her child’s eating and to encourage her child to pay more attention to the food being served by cutting the fruit into tiny pieces. Packaging was used by children beyond the core function of protecting its contents and has the potential to assist with play and learning.

#### Needs for packaging design solutions as portion control aids

Three main areas emerged from the video observations regarding mothers’ needs for packaging design solutions as portion control aids.

**Figure 7:**
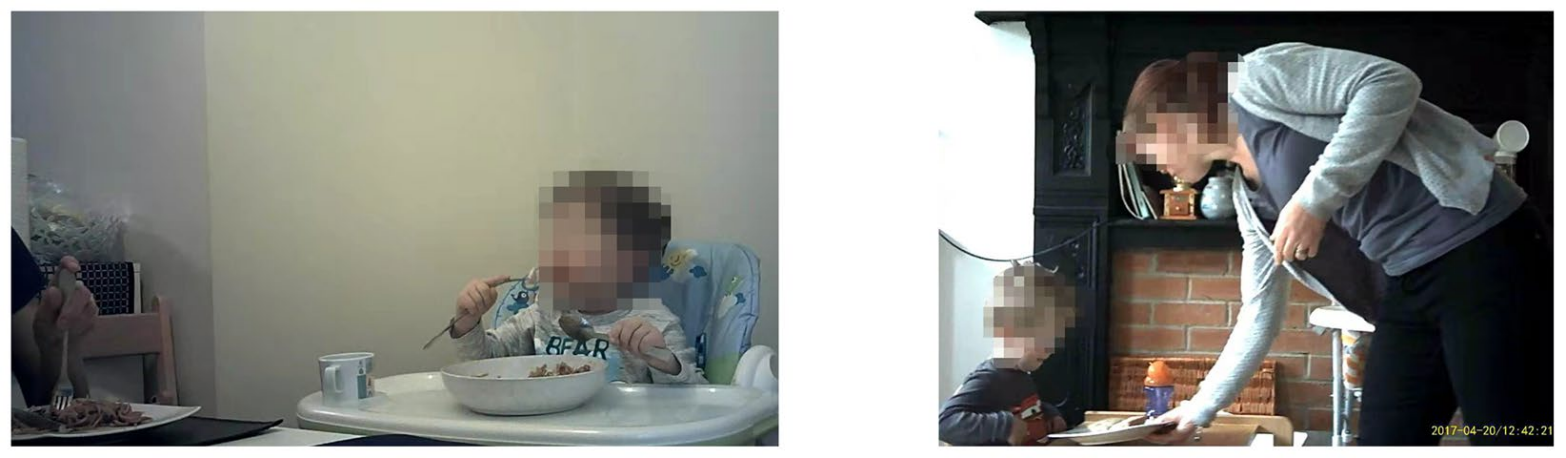
Use of standard “adult” size plates to offer children’s meals.

**Figure 8:**
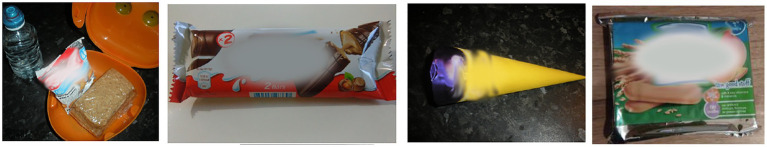
Pre-packaged snacks served to children. (1) MU01S1:48 m, (2) MUd03S:54 m, (3) MU01S1:48 m, and (4) MU03S:36 m.

**Figure 9:**
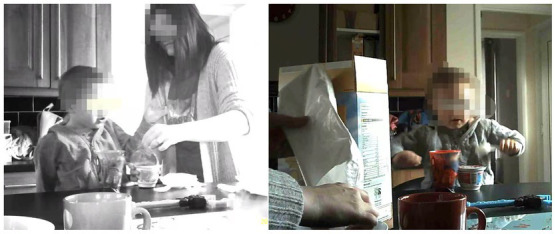
Pre-packaged adult size food offered to the child (MU04S2:49 m).

**Figure 10:**
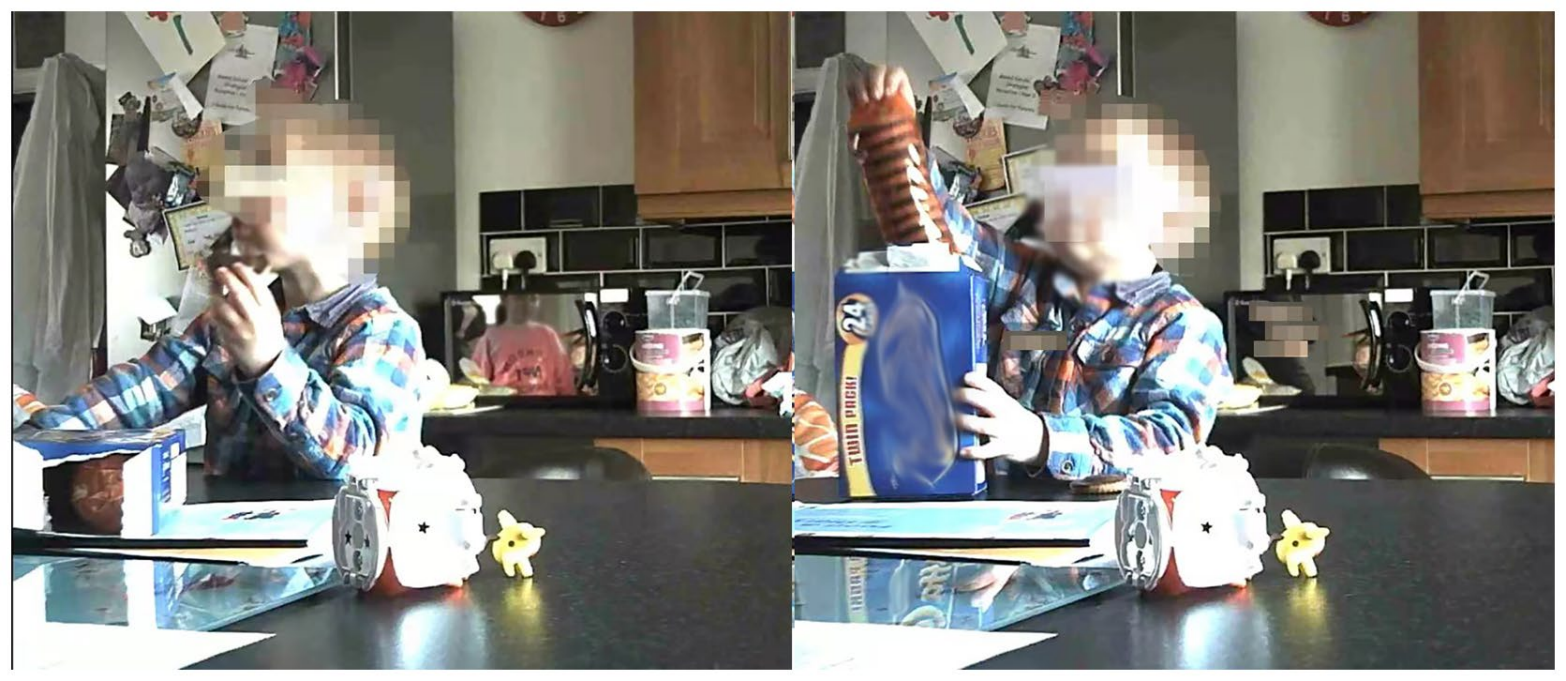
An example of a child serving themselves (MU04S2:49 m).

**Figure 11:**
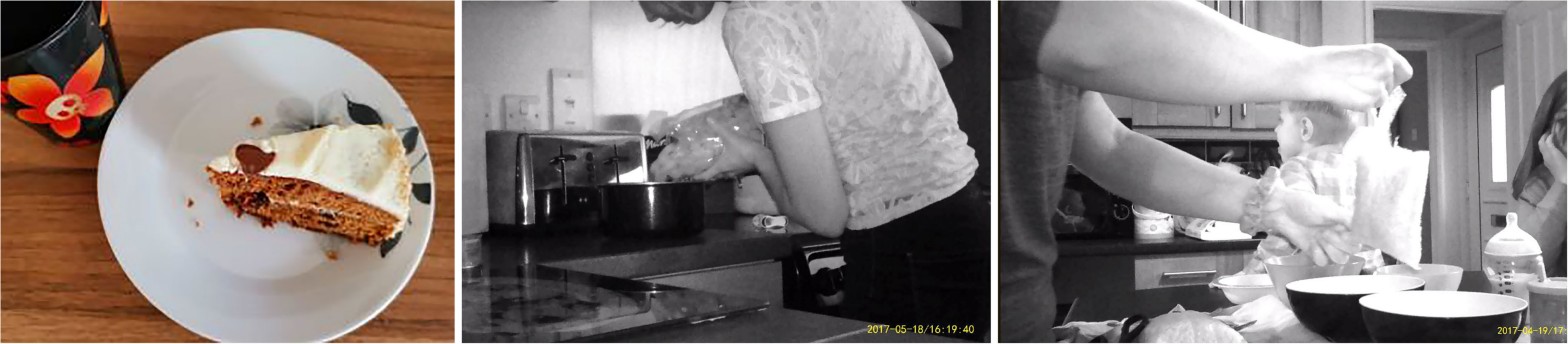
Determining the portion size by sight.

1. *Use of adult size dishware*: Parents tended to use plate sizes which were convenient and standard adult sizes. MU01 (Figure 7 right panel) served MU01S1:48 m meal items on a regular plate in a similar size that MU01 used for herself. MU02 served MU02S2:57 m sandwiches on a standard adult size plate for lunch.
2. *Size of pre-packaged snacks*: Since pre-packaged snacks are generally intended for adults, the household observations confirmed that most pre-packaged snacks were offered to children without modification for the age/stage of the child. Thus, children learn to accept standard/adult size portions as the norm. In Figure 8, a pre-packaged savoury snack (25 g; 123 kcal with 0.5 g salt) was packed in MU01S1:48 m’s lunchbox, a 43 g chocolate bar containing 17.8 g sugar was served to MUd03S:54 m; an ice-cream containing 16.9 g sugar and 205 kcal was served to MU01S1:48 m after lunch, and Heinz 7+ months biscotti cookies (60 g containing 13.2 g sugar) served to MU03S:36 m. According to NHS guidelines (2018), the maximum recommended sugar intake per day for 4–6 years is 19 g. In some cases, children helped themselves to pre-packaged snacks at home, and in other cases, mothers served adult size packaged foods to their children. For example, MU01S1:48 m ate a carton of yoghurt while his mother was cleaning up the dishes after dinner. MU04, at around 6 am, gave a pot of yoghurt in adult size (175 g) to their son MU04S2:49 m (Figure 9) while preparing breakfast for the family with three children. Figure 10 shows that MU04S2:49 m took and served himself two cakes from a Twin Pack of 20 cakes after dinner.
3. *Subdividing large portions by sight*: When subdividing from large portions of food items, decisions were made by sight without using a portion aid. Figure 11 left panel shows the portion served to MUd03S1:54 m from a large cake as a snack. Video footage illustrated when cooking pasta MU06 (Figure 11 middle panel) poured from the pack directly to the boiling water without weighing or using a scoop. MU04 split the steamed rice into children’s and adults’ bowls by sight without a specific measurement (Figure 11 right panel).

### Food diary results

**Figure 12:**
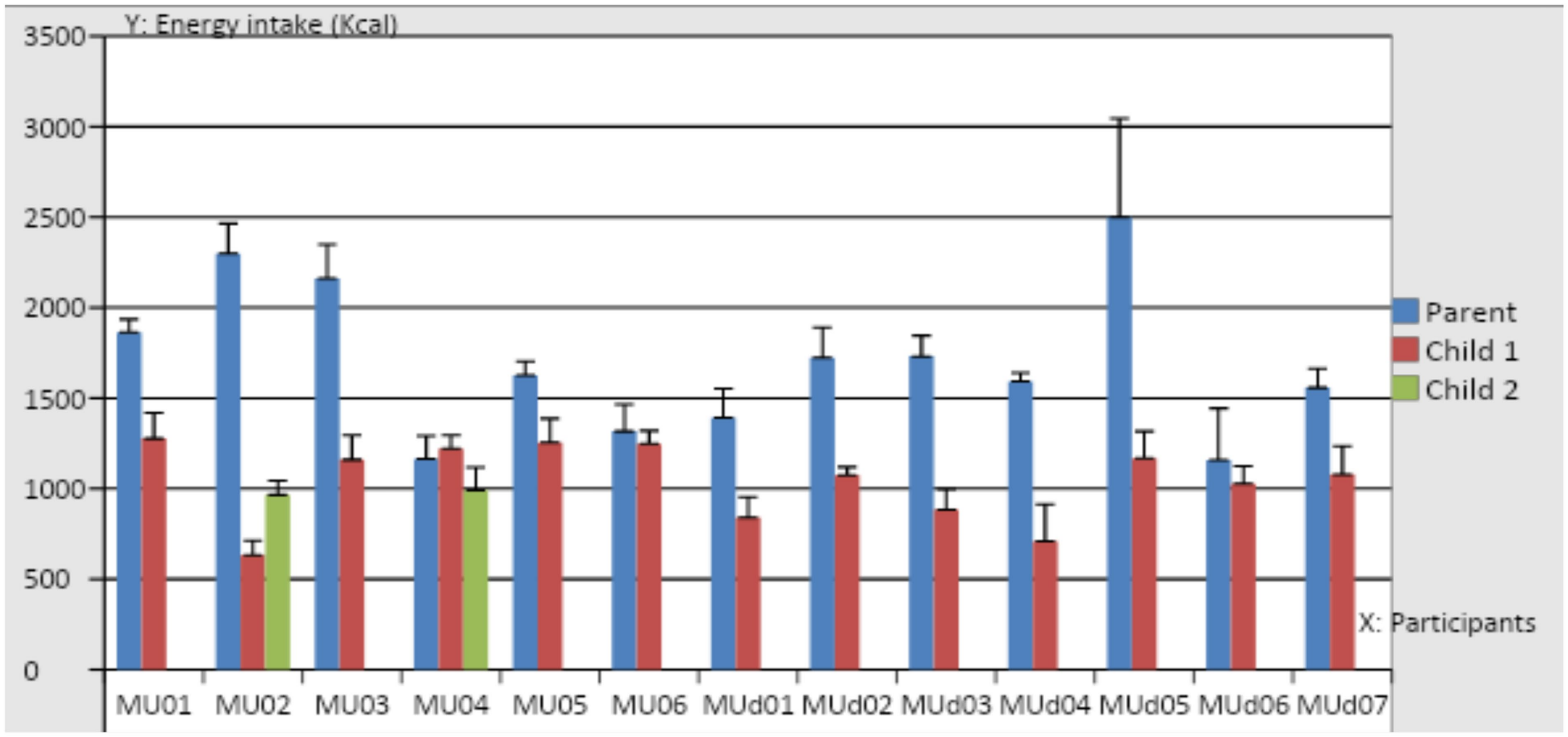
Mean (SD) daily energy intake of the participating mothers and children.

Figure 12 presents the mean daily energy intake (kcal) recorded over 4 days by 13 mothers and 15 children who were of preschool or reception age. Five child participants had higher total energy intake per day when compared to the British Nutrition Foundation recommendations for children this age. There were some significant correlations between types of foods that the mothers and children were eating, but children consumed more energy from snacks than their mothers. The mean daily energy intake consumed by the mothers during the study was 1699.8 kcal/day. 10.8% of energy came from the snacks (8.2%, 144.2 kcal from sweet snacks and 2.6%, 50.3 kcal from salty snacks; Supplementary Material D). The mean daily energy intake consumed by the children was 1,036 kcal/day. 15% of the energy came from the snacks (12.2%, 122 kcal from sweet snacks and 2.5%, 28 kcal from salty snacks). Six out of 13 mothers (MUd01, MUd02, MUd03, MUd05, MU01, MU04) offered their children more sweet snacks per day than they gave themselves (0 g for MUd04 and MUd05-81.00 g for MU03; 0 g for MUd04S, MUd07S and MU06D – 65.75 g for MU04S1). Four children exceeded 20% of their total energy intake per day from sweet snacks (MUd01D:28 m (27%), MUd03S1:54 m (23%), MU04S1:49 m (22%) and MU04S1:19 m (27%), whereas MU04S1:49 m exceeded this recommendation by 32% from salty and sweet snacks. This data provides a context within which it is possible to understand the general dietary intakes of the children, identifying where potential challenges lie (e.g., the number and amount of sweet and salty snacks consumed at home).

### Interview results

From the responses to interviews, a number of considerations about packaging emerged. These ranged from the ways that packaging influenced food preferences to adverse effects of packaging which encouraged intake of HED sweet and salty non-core foods.

#### Parents’ perceived effects of packaging on children

##### Shaping children’s taste perception and food preference

Mothers reported that food packaging is designed to be appealing to children, increasing their willingness to try unfamiliar foods and generally create favourable attitudes toward these products. The shape and graphical features (such as animal characters) of some packages were mentioned as affecting the children’s food product preference and improved interest in these foods:

“Before she (MUd05D: 25 m) did not like cheese biscuits, but once, she picked up one by herself because of its packaging. There was a bear on the packaging. So she starts to eat cheese biscuits” [MUd05].

“Yeah, sometimes, when the packaging is attractive to them. Take the yoghurt, for example, once I bought yoghurt in a strawberry-shaped pack, he (MUd06S1:16 m) liked it, and he ate it all, he liked the strawberry-shaped packaging” [MUd06].

##### Encouraging slow eating

Games printed on the packaging were considered as a means to influence their children at the moment of consumption. MU06 felt that packaging with the printed games grabbed her child’s attention and increased the consumption of healthy but less preferred foods. This echoed the observation of MU03’s (Figure 6 right panel) where a dry fruit packaging with an interactive game was used to encourage slower eating and less intake.

“I buy (dry) fruit snacks that come in small portions... They are animals’ paw shapes. He (MU03S:36 m) likes playing the matching game on the back of the packaging. He always takes one out and finds out whose the “paw” is before he eats it, even if he is hungry. It is a very small bag. The game slows down his eating otherwise, he would swallow them down very quickly and it helps him learn the animals and their paw shapes… if they are without the games on the packaging, he can eat two and three bags…” [MU03].

#### Concerns/negative perception and attitude towards packaging

##### Do not read portions

Mothers thought that informative packaging required time to read and comprehend, which would require time they did not have:

“I rarely read the portion. … I think most of the time the food that I am really used to, I have not read it” [MUi05].

“I probably do not look at the package to see what the portion size is. I do it least with cereal at breakfast time, because I definitely do not know (what) the portion size is for cereal. I do not measure breakfast cereal. Because cereal sizes are so small and he is really hungry at breakfast time. So I just let them have as much as they want” [MU05].

##### The use of cartoon characters on food packaging

This was considered a problem by mothers despite or, or because of, their appeal to children. Mothers thought that cartoon characters on some treat foods for children should be restricted to combat childhood obesity.

“It is very important to have a relevant design for the packaging design. These days you can see minions (characters from an animated movie) everywhere; children are crazy to see (them)” [MU01].

##### Environmentally friendly packaging

Mothers preferred environmentally friendly packaging, e.g., made with paper. When asked if they are or would be prepared to pay slightly more for foods that come in small portions, parents pointed out that buying small pre-packaged individual snacks and food items would cause more damage to the environment.

“Small packs aren’t sustainable. (It) does not make sense to me” [MUd03].

“Probably I disagree with that. It’s backwards. I understand why they do it, but it seems backwards to me” [MU05].

##### Practicality

Some mothers described prepackaged individual serving snack foods as favourable packaging features (e.g., MUi04, MUi05, MUd05). In contrast, others were concerned about the practicality of the suggested intervention, as “*different ages need (*packaging*) in different sizes*” (MUi08). Thus, downsizing interventions may succeed only if there is good fit with family lifestyle. Parents would not accept any time-consuming and effortful solutions if they affect everyday life, unless the gains from the improvement impact the health of their children.

“Informational and visual. Because the structural is difficult since different ages need (packaging) in different sizes” [MUi08].

### Needs for packaging as a downsizing solution

Table 3 summarises three specific areas where packaging design could be improved to meet mothers’ needs for packaging design as a downsizing solution, drawn from the interviews.

**Table 3:** Three types of creative packaging solutions for downsizing suggested by mothers.

Types	Quotations
Educational	“It would be better to see the information on the packaging which compares the weak child on an unhealthy diet and the strong child on a healthy diet. So using images shows children directly and children might be quick to learn which is good and which is bad for their health. It’s more effective than just telling them off” [MUi04]. “For unhealthy food, icons or symbols should be designed to make sense to the children. Parents could know how much is appropriate for children to have. Traffic lights are for adults and we do not understand” [MUd03]. “I think it would be nice for my older daughter who is eight to be able to read the packaging by herself. She can learn to be a healthy eater. My daughter (MU05D:7y5m) started to ask questions like how you know if I have an ok lunch today. Maybe she is thinking about how I eat healthy at eight” [MU05].
Interactive	“We just brought out some crisps made from…like parsnips that they do not expect from you. One of the packs has a maze on the back of it, and my daughter ate all the crisps… because she was doing the maze on the back. It was interesting to her” [MU05]. “They are not involved in cooking, but when they do it will become more interesting” [MU02]. “…Measuring food is educational; tools in different shapes make a collection” [MUd03].
Autonomy – independent use	“Visuals are easy for kids to be engaged with. Children are able to see and decide” [MU04]. “The packaging should be easy for kids to open by themselves” [MUd06]. “Maybe small games on the packaging can be educational… telling them how much they should eat per day and encouraging them to make decisions themselves” [MU03].

### Mismatch between household observation and reported behaviours

Household observations did not match what was reported in interviews. However, the videorecordings represent only a snapshot of home usage of packaging.

In the interviews, mothers reported use of portioning aids, including using small containers, e.g., child-sized bowls (MU06, MUd02, MUd03, MUd04, MUd07, MUi04, MUi05), plates (MU01, MU04, MU05, MUd03) and spoons (MU03 and MUd04), hand measurements (MU05, MUd07), and serving spoons and scale (MUd04) and they adjusted portions of HED snacks to provide ‘something small’ for their children (MU01), e.g., halving pre-packaged foods to their children (e.g., MU05, MUd02; MUd06).

“...never, savoury snacks - once a day, confectionery and cakes – I give them Kid size … I would not let them have the same size as me … just being logical” [MU02].

Household observations demonstrated that adult size plates (e.g., MU01, MU02) were used to serve meals to young children. Also, adult size pre-packed snacks (e.g., MU01, MU04, MUd03) were offered to young children without modification. MU01S1:48 m and MU04S2:49 m were served yoghurt cartons in adult sizes. Of interest, is that MU04 reported feeling confident about serving age-appropriate portion sizes. In the interview, she described that she used child-size plates to measure the right size (of meals) and gave her children snacks but was “not sure about correct portion sizes” (MU04). Combined with the demographic information from user profile questionnaires, we found that parents, especially those working full-time (e.g., MU03; MU04) or having two and more children (e.g., MU01; MU02; MU04) offered pre-packaged individual snacks for convenience and to save time.

The snacking observation showed that one of the mothers reused a 7-month baby food jar to determine the amount of homemade fruit yoghurt to give to her child MU03S:36 M (Figure 2 middle and right panels). During the final interview, MU03 stated that after filling out the food diary, she checked the Infant and Toddler Forum portion size table provided by this study. She discovered that the use of the meal jar for portioning yoghurt far exceeded the portion sizes of yoghurt recommended for children aged 2–4 years. This underlines the need to have information available on pack for children’s portions.

In addition, food diary analysis revealed that five out of 13 mothers did not downsize sweet snacks for their children. Four out of 15 children consumed savoury and sweet snacks which constituted between 21–32% of total daily energy consumption. The food diary analysis reveals that the average sweet snack intake for MU02S1:23 m accounted for 19% of his total energy consumption. It remains unclear what constitutes a “small” amount and why some parents are not implementing restrictions, guidance and recommendations they mentioned in the interviews.

### Impacts of the lack of portion control strategies

Mothers recognised large portions and realised that this might increase intake, but they may have underestimated the size of this difference, an observation that has been reported before (Reale et al., 2019a,b). When subdividing from large portions of food items, decisions were made by sight without using a portion aid in the observational studies. For example, Figure 11 left panel shows the portion served to MUd03S1:54 m from a large cake as a snack. The child food diary analysis reveals that the average sweet snack intake for MUd03S1:54 m accounted for 23% of total energy consumption.

It was evident when children served themselves they served large portions of HED foods that were highly appealing. MU04S2:49 m took two cakes in the home observation (Figure 10). Each small cake (12.2 g) contains 6.4 g sugar and 46 kcal energy. Two Jaffa cakes consist of two-thirds of the maximum recommended sugar intake per day for 4–6 years (19 g) by World Health Organisation (WHO) (2015) guidelines. Food diary results in Supplementary Material D showed that 21.55% daily energy intake of MU04S2 was from high sugar snacks.

## Discussion

In this work, we applied a mixed-methods approach to study the way that mothers and their young children used packaging for portion control in the home environment and to uncover the mothers’ needs for packaging design to support age appropriate portion sizes. Food diaries and home-based observations revealed discrepancies between what was said and what was done. The problem of large portion sizes was recognised and reported, but ways to downsize portions were not necessarily applied at home. Interviews revealed an incongruity between reporting the use of portion size aids such as smaller containers and dishware, and the actual size of plates used during video observations. Also, parents reported that portions were made smaller for children, but when children served themselves they generally did not downsize amounts served as reflected in both video observations and food diaries. Therefore, the easiest nudge for parents is to provide physically smaller plates, bowls and prepackaged foods since mothers relied on environmental cues to guide to serving size. Interviews provided evidence of interest and motivation in downsizing portions of HED foods with solutions to improve packaging using educational and interactive ways to facilitate autonomy for age-appropriate portion sizes. Mothers expressed the need for better education and engagement as important parameters of packaging design to downsize portions for their children.

This study’s observational findings illustrated the mothers’ reliance on previous experience, visual estimation and child self-regulation to decide on amounts of foods to offer at home, but none used measurement aids, made reference to nutrition guidelines, nor expert recommended portion sizes for children. This confirms previous research that portioning is highly influenced by contextual factors, including package size and child requests (Blake et al., 2015). However, Almiron-Roig et al. (2013) demonstrate that adults remain unaware of reference portion sizes, which may facilitate overconsumption. Mothers may misjudge portion sizes for children, which could be influenced by perceptual error, particularly when faced with large portions (Hetherington et al., 2018). The present study provides further evidence illustrating that nutrition education, especially appropriate aids, guidance and serving size adjusted for child age and stage is needed at the point of serving, which is necessary given the potential for parental perceptual error (Almiron-Roig et al., 2013; Reale et al., 2019a,b). Furthermore, this investigation noted that mothers were enthusiastic about learning portion control from their participation in the research. To respond to the needs identified, innovative packaging design and creative packaging prototypes to downsize portions of HED foods for children should be developed and then tested in home environments.

In the present study, children were engaged with mazes and games embedded on food packaging, suggesting that packaging has the potential to assist downsizing through play and learning. This supports findings by Enax et al. (2015) - that fun, child-directed packaging is rated highly in terms of taste perception, and children find the food attractive. The data from this study illustrates that mothers called for child-directed packaging to offer HED foods in small sizes. Research by de Droog et al. (2017) demonstrates the use of hand puppets to involve children in interactive reading to promote vegetable consumption. Therefore, fun and interactive child-friendly packaging design might offer opportunities to attract children to healthy eating, learn about portion control and establish portion norms through enhanced playful engagement and narrative development (Tang et al., 2022).

It was observed that children helped themselves to foods from adult-sized or family-sized packaging at home, especially when mothers/caregivers were busy. However, children may not recognise when they are full (Fisher et al., 2007) and are just beginning to learn about appropriate portion size for foods which are highly liked. With repeated exposure to adult size portions, they may become familiar with such large portion sizes and these become normalised (Robinson et al., 2016). Given that many parents relied on the size of pre-packaged foods as a guide to appropriate portion sizes, more effortless and child-friendly packaging solutions are needed to encourage greater autonomy for self-serving even when the food is tempting and highly liked. Packaging design would constrain the portion sizes through a behavioural steer (Jelsma and Knot, 2002; Bhamra et al., 2011) - the physical characteristics of the product encouraging children to downsize in ways prescribed by the designer.

However, the use of the games and mazes on the packaging may cause a distraction from eating. Therefore, creative packaging design might provide interactivity by raising awareness of the food texture and the amount that is eaten as well as influencing the speed of eating.

The mixed methods approach was a strength in this study since real time product use was observed alongside self-report, uncovering some unexpected use of the products by children themselves, identifying the unmet needs of the design-led solutions for downsizing palatable HED food and drink items in children. Given the habitual nature of food provisioning (Chandon, 2013; Tang et al., 2020), the home-based video recordings expand details of everyday practices and mundane interactions between the users and packaging products which people may not report, nor be able to articulate when asked in interviews.

However, there are methodological limitations which constrain generalization beyond this sample. The parents generally had a relatively high social status (education, income) and a strong motivation for participating in research. Scores on four out of 12 CFPQ constructs differed from published norms (Musher-Eizenman and Holub, 2007). The child sample was dominated by boys (17/25). Additionally, the sample size for the food diaries is insufficient to test any statistical relationships from the data. Clearly, these limitations constrain the findings. However, since results confirm and extend previous research and have the added benefit of home-based observation, this adds to the literature. In particular, a strength of the study was the ways that packaging was used at home and the identification by mothers of how packaging design could be improved through the triangulation between three research methods. The findings require further theoretical investigation and empirical studies to be conclusive.

Mothers reported the need for manufacturers to provide better visual representation, prepackaging and support for portion size. Implications for food industry include making packaging more informative, educational, and engaging to support child-friendly portion size, through awareness, play, interaction and autonomy. For mothers, nutrition education and especially implementing appropriate aids, guidance and serving size adjusted for child age and stage is needed. To respond to the needs identified, food manufacturing should assist parents through innovative packaging design and creative packaging prototypes to meet family needs which can be trialed in home environments.

## Data availability statement

The original contributions presented in the study are publicly available. This data can be found at: https://osf.io/m6syt/.

## Ethics statement

The studies involving human participants were reviewed and approved by University of Leeds Faculty Ethics committee (ref PVAR 15-096). Written informed consent to participate in this study was provided by the participants' legal guardian/next of kin.

## Author contributions

TT, WW, and MH designed the research, analysed and interpreted the data, and wrote the manuscript. TT, WW, MV, and FC collected the data. FC analysed all diet diaries. All authors contributed to the article and approved the submitted version.

## Funding

This study was supported by BBSRC grant BB/M027384/1 (Downsizing: using environmental cues to acquire healthy portion control in children, adolescents and their families).

## Conflict of interest

The authors declare that the research was conducted in the absence of any commercial or financial relationships that could be construed as a potential conflict of interest.

## Publisher’s note

All claims expressed in this article are solely those of the authors and do not necessarily represent those of their affiliated organizations, or those of the publisher, the editors and the reviewers. Any product that may be evaluated in this article, or claim that may be made by its manufacturer, is not guaranteed or endorsed by the publisher.

## Footnotes

^1^ https://www.nutrition.org.uk
