# Supplementary material for: Designing for downsizing: Home-based barriers and facilitators to reduce portion sizes for children
Source: Front Psychol. 2022 Oct 3;13:915228. doi: 10.3389/fpsyg.2022.915228 (PMC9575649; doi:10.3389/fpsyg.2022.915228)
Supplement: Supplementary file 1 [file Data_Sheet_1.PDF]

frontiers

Supplementary Material A

User profile questionnaire

Section A - Food Shopping (Please tick the appropriate box)

1. What is your level of responsibility for household food shopping?
   - a. Not responsible ☐
   - b. Less than a half ☐
   - c. About a half ☐
   - d. Almost all ☐
   - 1-1. Who else is responsible for food shopping in your household? ______

2. How often do you (or someone else) do a main shop for food shopping?
   - a. Everyday ☐
   - b. 2-3 times a week ☐
   - c. About once a week ☐
   - d. 2-3 times a month ☐
   - e. Once a month ☐
   - f. Less than once a month ☐
   - g. Never ☐

3. Where do you (or someone else) do your main food shopping?
   - a. Supermarket ☐
     - a-1. Please specify the name of the supermarket ______
   - b. Grocery stores/specialist shops ☐
   - c. Markets ☐
   - d. Internet ☐
   - e. Other (Please specify) ______

4. What types of food do you usually buy for your main food shopping?
   - a. Meat, fish and alternatives ☐
   - b. Fruit and vegetables ☐
   - c. Confectioneries ☐
   - d. Savoury snacks ☐
   - e. Cereals ☐
   - f. Bakery ☐
   - g. Dairy products ☐
   - h. Frozen food ☐
   - i. Wine, champagne and beer ☐
   - j. Sugary drinks ☐
   - k. Tea, coffee and juice drinks ☐
   - l. Other (Please specify) ______

Section B - Eating Habits

1. When and where do you eat breakfast?<br><br>2. When and where do you eat dinner?<br><br>3. When and where does your child often eat snacks?

Section C- You and Your family

**Your information**				
Age (in years)				
Occupation				
Your height	cm OR feet (ft) and inches			
Your weight	kg OR stone lbs			
Your ethnic group	(Please tick the appropriate box)			
	White	Black	Asian	Mixed
	White British ☐	Caribbean ☐	Indian ☐	White and Blac Caribbean ☐
	White Irish ☐	African ☐	Chinese ☐	White and Blac African ☐
			Pakistani ☐	White and Asia ☐
			Bangladeshi ☐	
	Other White ☐	Other Black ☐	Other Asian ☐	Other Mixed ☐
	Please specify	Please specify	Please specify	Please specify
Your highest education level	a. No qualifications ☐<br>b. CSE, GCSE or O-level ☐<br>c. Vocational qualification (GNVQ or BTEC) ☐<br>d. Higher national certificate or Diploma ☐<br>c. Undergraduate degree ☐<br>d. Postgraduate qualification (master/PhD) ☐			
Your work status	a. Full-time ☐<br>b. Part-time ☐<br>c. Not working/retired ☐			
Family structure	a. Two-parent/caregiver family ☐<br>b. Single parent ☐			
Average household income	a. £0-£10,000 ☐<br>b. £10,000-£20,000 ☐<br>c. £20,000-£30,000 ☐<br>d. £30,000-£40,000 ☐<br>e. £40,000+ ☐			
**Your partner's information**				
Age (in years)				
Occupation				

Height	cm OR feet (ft) and inches
Weight	kg OR stone lbs
Your partner's ethnic group	(Please tick the appropriate box)

White	Black	Asian	Mixed
White British ☐	Caribbean ☐	Indian ☐	White and Blac Caribbean ☐
White Irish ☐	African ☐	Chinese ☐	White and Blac African ☐
		Pakistani ☐	White and Asia ☐
		Bangladeshi ☐	
Other White ☐	Other Black ☐	Other Asian ☐	Other Mixed ☐
Please specify	Please specify	Please specify	Please specify

Your partner's highest educatio level	a. No qualifications ☐<br>b. CSE, GCSE or O-level ☐<br>c. Vocational qualification (GNVQ or BTEC) ☐<br>d. Higher national certificate or Diploma ☐<br>c. Undergraduate degree ☐<br>d. Postgraduate qualification (master/PhD) ☐

**Your children's information**		
1	Gender	
	Date of birth	
	Height	_____ centimetres (cm) OR _____ feet (ft) and _____ inches
	Weight	_____ kg OR _____ stone _____ lbs
2	Gender	
	Date of birth	
	Height	_____ centimetres (cm) OR _____ feet (ft) and _____ inches
	Weight	_____ kg OR _____ stone _____ lbs
3	Gender	
	Date of birth	
	Height	_____ centimetres (cm) OR _____ feet (ft) and _____ inches
	Weight	_____ kg OR _____ stone _____ lbs

Thank you for answering this questionnaire for the project.

Supplementary Semi-structured interview guide

The interviews were conducted by the authors (TT, WW, MV) who agreed a course of action, to put participants at their ease and to read out each question in turn, with the opportunity to address any

questions they had about this interview. The guide consisted of six sections including 44 questions that explored more in-depth about the parents' perceptions of portion size, child-parent purchase relationship, feeding practices and portion size strategies, their intention and confidence (self-efficacy beliefs) to serve age appropriate portions of meals and snacks, and parents' perceptions and needs of packaging solutions for downsizing.

A. Parents' perceptions of portion size

1. Are you concerned about your weight?
2. Are you concerned about your child's weight?
3. What does "eating healthy" mean to you?
4. Do you think it is important for your child/children to acquire the habit of eating healthily from young?
5. Do you control portions? If so, how do you decide how much you eat?

B. Child-parent purchase relationship

6. What factors do you consider most when you buy foods for your child(ren) aged 1-5 years old?
7. Does your child try to influence you when you do grocery shopping?
8. How does your child often influence your purchasing decision?

Below are statements regarding how packaging influences your child(ren)'s preference for food and snacks. Please select the statement that most closely applies to you and give some examples.

	Strongly Disagree	Disagree	Neutral	Agree	Strongly Agree	Any examples
9. My child's product preferences are influenced by the packaging.						
10. My child's product preferences are influenced by cartoon characters on the packaging, e.g. Peppa Pig						
11. My child's product preferences are influenced by packaging which is an interesting shape						
12. My child's product preferences are influenced by offers of free gifts displayed on packaging						
13. My child's product preferences are influenced by bright interesting and attractive colours used in packaging						
14. My child's product						

preferences are influenced by the brand which she/he is already familiar with.					
15. I am or would be prepared to pay slightly more for foods that come in small portions					
16. I just want to go out and buy whatever food my child(ren) likes without being bothered by portion control messages					

17. When you do grocery shop with your child, how you often resolve the conflicts or reach to a consensus with your child?

C. Feeding practices and portion size strategies

18. Is your child difficult to feed?
19. How do you get your child to eat food that they don't like/healthy food? E.g. vegetables?
20. How do keep your child from eating too much HED/junk food?
21. Do you allow your child to eat breakfast cereals? Savoury snacks? Confectionery (e.g. cakes, cookies, ice cream? Sugar sweetened drinks?
22. When do you allow your child to eat breakfast cereals? Savoury snacks? Confectionery (e.g. cakes, cookies, ice cream? Sugar sweetened drinks?
23. How much (breakfast cereals? Savoury snacks? Confectionery (e.g. cakes, cookies, ice cream? Sugar sweetened drinks?) is your child allowed to eat?
24. How do you decide how much of a food to offer to your child?
25. How do you control or balance the amount of food your kid likes - and might be unhealthy - and food he/she dislikes – but is healthy?
26. What are your main concerns when purchasing and serving food for your child?

D. Intention to serve child-sized portions and downsize HED foods

27. Have you ever heard about 'me size meals/snacks' or 'kid-sized meals/snacks'?

No. (Stage 1:unaware of Issue)

Yes. (Go to **28**]

28. How did you hear about this?
29. Have you served age appropriate portion of the meals/snacks for your child(ren)?

No. [Go to 30]

Yes. (Stage 6: acting; Go to 31]

30. Which of the following best describes your thoughts about serving 'me size meals' or 'kid-sized meals/snacks'? And why do you decide to do so?

SHOW CARD with following options

A) I've never thought about doing this.	(Stage 2:unengaged by issue)<br>(Stage 3:undecided about

B) I'm undecided about doing this.<br>C) I've decided I don't to want to do this.<br>D) I've decided I do want to do this.	acting)<br>(Stage 4: decided not to act)<br>(Stage 5: decided to act)

31. how long have you been serving kid-sized meals/snacks?

A) Less than 6 months

B) 6 months or more

32. On the whole how do you feel that you've been doing? Explain why?

E. Confidence (self-efficacy beliefs) to serve age appropriate portions

SHOW CARD with following options

A) Not at all confident<br>B) Somewhat confident<br>C) Confident<br>D) Very confident

33. How confident are you that you are able to serve your child age appropriate portion sizes of meals at home? Being confident means that you know that you can do something.
34. How confident are you that you are able to serve your child age appropriate portion sizes of snacks at home? Why?
35. How confident are you that you are able to serve your child age appropriate portions of meals when you are eating meals and snacking away from home? Why?
36. How confident are you that you are able to serve your child age appropriate portions of snacks when you are eating meals and snacking away from home? Why?
37. How confident are you about the things can give your child as a healthy snack? Why?
38. How confident are you about the other things that you can give your child as a reward instead of a snack? Why?

F. Parents' perceptions and needs of packaging solutions for downsizing

39. Does packaging affect your decisions concerning purchasing foodstuffs for your child? If so, how does it affect your purchase?
40. How could the packaging design be improved to help your child/children develop healthier eating habits?
41. Do you think current packaging helps you decide how much of a food to offer to your children of different ages?
42. How could the packaging can be improved to help you decide how much of a food to offer to your child? Explain why?
43. Would you like to have more information on age appropriate portion sizes? If so, what types of products/supports (Messages? Application? Services?) would help you decide the amount of food to offer to your child? Explain why?
44. Any comments on the packaging for child's food and drinks?

Supplementary Material B

Supplementary data

Mother IDs and child IDs

**Participant code**	**Child aged 1-5 years**	**Age in months**	Child outside the age range of 1-5 years (age)
MU01	MU01S1	48m	MU01S2* (8y9m)
MU02	MU02S1<br>MU02S2	23m<br>57m	
MU03	MU03S	36m	
MU04	MU04S1<br>MU04S2	19m<br>49m	MU04D* (8y)
MU05	MU05S	58m	MU05D*(7y5m)
MU06	MU06D	56m	
MUd01	MUd01D	28m	MUd01S (7y）
MUd02	MUd02S	27m	MUd02D (5y9m)
MUd03	MUd03S1	54m	MUd03S2 (1m)
MUd04	MUd04S	58m	
MUd05	MUd05D	25m	
MUd06	MUd06S1<br>MUd06S2	16m<br>54m	
MUd07	MUd07S	35m	MUd07D (8y)
MUi01	MUi01S1;<br>MUi01S2	51m<br>51m	
MUi02	MUi02D	31m	
MUi03	MUi03D	29m	
MUi04	MUi04D	56m	MUi04S (6y9m)

MUi05	MUi05D	27m	MUi05S* (5y10m)
MUi06M	MUi06D1	44m	MUi06D2 (5m)
MUi07M	MUi07S	20m	
MUi08M	MUi08S1	25m	MUi08S2 (5y1m)

Participant characteristics

		All		Group1		Group2		Group3	
	**Attribute**	**Tot**	**%**	**Tot**	**%**	**Tot**	**%**	**Tot**	**%**
**Mother**				MU01-MU06		MUd01-MUd07		MUi01-MUi08	
Age (years)	21-30	3	14%	2	33%	1	14%		
	31-40	16	76%	4	67%	4	58%	8	100%
	41-50	1	5%			1	14%		
	51-60	1	5%			1	14%		
Age range			24-51		24-39		30-51		31-39
Mean age			35.1		33		37.9		34.5
BMI (kg/m^2)**	Underweight	2	10%			1	14%	1	12.5%
	Normal weight	15	71%	5	83%	4	57%	6	75%
	Overweight	4	19%	1	17%	2	29%	1	12.5%
Ethnicity	White British	11	52%	4	66%	4	57%	3	38%
	African	1	5%					1	12%
	Other white/white Irish	1	5%	1	17%				
	Chinese	8	38%	1	17%	3	43%	4	50%
Highest Education	Vocational qualification (GNVQ or BTEC)	2	10%	1	17%			1	13%
	Higher national certificate or Diploma	5	24%			3	43%	2	26%
	Undergraduate degree	6	28%	2	33%	1	14%	3	35%
	Postgraduate qualification (master/PhD)	8	38%	3	50%	3	43%	2	26%
Employment Status	Full time	5	24%	2	33%			3	38%
	Part-time	8	38%	2	33%	3	43%	3	38%
	Not working	8	38%	2	33%	4	57%	2	24%
Marital Status	Two-parent/caregiver family	20	95%	6	100%	7	100%	7	88%
	Single parent	1	5%					1	12%
Income	£10–20,000	4	19%	1	16%	2	28.5%	1	13%
	£20–30,000	4	19%	1	16%	1	14%	2	26%
	£30–40,000	7	33%	2	34%	2	28.5%	3	35%
	£40,000+	6	29%	2	34%	2	28.5%	2	26%
**Child**									
Sex	Female	8	32%	1	13%	2	25%	5	56%
	Male	17	68%	7	87%	6	75%	4	44%

Age (months)	12-24	4	16%	2	25%	1	12%	1	11%
	25-36	9	36%	1	12.5%	4	50%	4	45%
	37-48	2	8%	1	12.5%			1	11%
	49-60	10	40%	4	50%	3	38%	3	33%
BMI Centile*	Overweight	5	20%	2	25%	2	25%	1	11%
	Normal weight	18	78%	6	75%	5	62%	7	78%
	Underweight	2	8%			1	13%	1	11%

* Body mass index (BMI) centile based on Boys & Girls UK-WHO Growth Chart 0-4years, Boys & Girls UK body mass index 2-20 and child's height and weight reported by parents. Overweight is defined as >91st centile and underweight < 2nd centile.

** BMI calculated from self-reported height and weight and classified as underweight <18.5 kg/m^2; normal weight 18.5–24.9 kg/m^2; overweight 25–29.9 kg/m^2; obese >30 kg/m^2.

Supplementary Material C

Supplementary data

CFPQ results

	Mean±SD	Mean±SD (Musher-Eizenman et al. Table 1)	p	Range	Mean±SD of group 1	Mean±SD of group 2	Mean±SD of group 3
Restriction for Weight Control	2.2±0.7	1.6±0.9	0.1903	1.0 - 3.6	2.1±1.1	2.3±0.6	2.1±0.5
Restriction for Health	4.0±0.8	2.9±1.4	0.0094	2.0 - 5.0	4.0±0.7	4.3±0.7	3.8±0.9
Pressure	3.2±0.7	2.3±1.3	0.0308	1.5 - 4.0	3.4±0.4	3.5±0.6	2.7±0.7
Food as a Reward	2.8±1.0	2.1±1.3	**0.0005**	1.0 - 5.0	2.7±0.8	2.8±1.4	2.8±0.9
Emotion Regulation	2.4±0.6	1.7±0.7	**0.0038**	1.3 - 3.3	2.3±0.7	2.5±0.4	2.3±0.7
Monitoring	4.1±0.7	4.4±0.7	0.2553	2.5 - 5.0	4.2±0.8	4.0±0.9	4.2±0.6
Modelling	4.4±0.7	4.4±0.9	0.9032	2.5 - 5.0	4.6±0.5	4.6±0.20	4.0±1.0
Environment	4.1±0.7	3.9±1.0	0.2762	2.5 - 5.0	3.9±1.0	4.3±0.5	4.0±0.6
Child Control	2.9±0.6	2.8±1.0	0.6945	1.6 - 3.8	2.5±0.7	3.1±0.5	3.0±0.6
Teaching about Nutrition	4.4±0.7	4.2±1.1	0.6757	2.7 - 5.0	4.3±0.6	4.2±0.9	4.5±0.6
Child Involvement	4.0±0.9	3.8±1.7	0.6945	2.0 - 5.0	3.8±0.8	4.0±1.1	3.8±1.3
Encourage Balance and Variety	4.6±0.4	4.6±0.6	0.9483	4.0 - 5.0	4.6±0.4	4.5±0.4	4.5±0.6

Supplementary Material D

Supplementary data

Supplementary Table 1 comparing average energy intake per day of parent participants in the food diary studies with the BNF recommendations

	One-Sample Statistics				One-sample Test						
	N	Mean per day	Std.Deviation	Std.Error Mean	Test Value	t	df	p.(2-tailed)	Mean Difference	95%Confidence Interval of the difference	
										Lower	Upper
MUd01	4	1393	320.536	160.268	2103	-4.43	3	0.021	-710	-1220.04	-199.956
MUd02	4	1723.5	334.1721	167.0861	2103	-2.271	3	0.108	-379.5	-911.242	152.2424
MUd03	4	1731.75	227.1525	113.5762	2103	-3.269	3	0.047	-371.25	-732.7	-9.79971
MUd04	4	1594	91.5678255	45.78391275	2103	-11.117	3	0.002	-509	-654.704844	-363.295156
MUd05	4	2500	1089.723	544.8615	2175	0.596	3	0.593	325	-1408.99	2058.992
MUd06	4	1158.5	571.2609	285.6304	2175	-3.559	3	0.038	-1016.5	-1925.5	-107.496
MUd07	4	1558.75	206.7258652	103.3629326	2103	-5.265	3	0.013	-544.25	-873.1969829	-215.3030171
MU01	4	1865.5	140.7160261	70.35801305	2103	-3.376	3	0.043	-237.5	-461.4105987	-13.58940133
MU02	4	2299	330.3765528	165.1882764	2103	1.187	3	0.321	196	-329.70282	721.70282
MU03	4	2162.75	374.3958467	187.1979233	2103	0.319	3	0.771	59.75	-535.9973395	655.4973395

MU04	4	1164.75	255.4060493	127.7030246	2175	-7.911	3	0.004	-1010.25	-1416.658019	-603.8419811
MU05	4	1627.75	150.3936501	75.19682507	2103	-6.32	3	0.008	-475.25	-714.5598581	-235.9401419
MU06	4	1317.75	293.9596061	146.979803	2175	-5.832	3	0.01	-857.25	-1325.005331	-389.4946689

Supplementary Table 2 comparing average energy intake per day of children participants in the food diary studies with the BNF recommendations

	One-Sample Statistics				One-sample Test						
	N	Mean	Std.Deviation	Std.Error Mean	Test Value	t	df	p(2-tailed)	Mean Difference	95%Confidence Interval of the difference	
										Lower	Upper
MUd01D	4	841	226.60244	113.30122	980	-1.227	3	0.307	-139	-499.5751	221.5751
MUd02S	4	1075	88.47975	44.23988	1046	0.656	3	0.559	29	-111.791	169.791
MUd03S	4	885	224.53656	112.26828	1434	-4.89	3	0.016	-549	-906.2878	-191.7122
MUd04S	4	709.5	409.2566432	204.6283216	1466	-3.697	3	0.034	-756.5	-1407.718646	-105.281354
MUd05D	4	1168	297.8534315	148.9267158	944	1.504	3	0.23	224	-249.951276	697.9512763
MUd06S	4	1027.5	191.5646105	95.78230526	845	1.905	3	0.153	182.5	-122.322043	487.3220435
MUd07S	4	1079.25	156.4765691	78.23828453	1157	-0.994	3	0.394	-77.75	-326.7391395	171.2391395
MU01S	4	1277.5	283.8056847	141.9028423	1017	1.836	3	0.164	260.5	-191.0981763	712.0981763
MU02S1	4	633.5	160.4794899	80.23974493	984	-4.368	3	0.022	-350.5	-605.8586798	-95.14132021
MU02S2	4	966.75	155.6928065	77.84640326	1458	-6.311	3	0.008	-491.25	-738.9919984	-243.5080016
MU03S	4	1160.75	269.613767	134.8068835	1224	-0.469	3	0.671	-63.25	-492.2656683	365.7656683
MU04S1	4	1223	145.0080458	72.50402288	1394	-2.358	3	0.1	-171	-401.7401597	59.74015972
MU04S2	4	992.5	252.2941405	126.1470703	904	0.702	3	0.533	88.5	-312.9562777	489.9562777

MU05S	4	1255.75	261.7115142	130.8557571	1466	-1.607	3	0.206	-210.25	-626.6914207	206.1914207
MU06D	4	1249.5	142.2591532	71.1295766	1338	-1.244	3	0.302	-88.5	-314.8660582	137.8660582

Supplementary Table 3 Average daily consumption of the parent and child participants in the food diary studies

Code		Fruit (g)	Vegetables (g)	Savory snacks (g)	Sweets (g)	Chocolate (g)	Cakes/biscuits (g)	Ice-cream (g)	Sugar Sweetened Beverages (g)	Average energy intake (kcal)	Savory snacks (kcal)	% E from Savory snacks	Total sugary snacks (g)	Sugary snacks (kcal)	% E from sweet snacks	Average number of HED snacks*
MUd01	Parent	82	79.25	0	0	25	0	0	0	1393	0	0%	6.25	32.50	2.33%	
MUd01D	Child 1	77	17.5	0	0	105	15	96	0	841	0	0%	54	230	27.35%	2.75
MUd02	Parent	161.3	119.5	25.25	0	0	20	0	62.5	1723.5	128.00	7.43%	5.00	20.75	1.20%	
MUd02S	Child 1	182.5	66.25	9.25	0	0	153	0	107.5	1075	46.75	4.35%	38.25	165	15.35%	1.5
MUd03	Parent	287.5	305	0	0	0	100	0	0	1731.75	0	0%	25.00	103.25	5.96%	
MUd03S	Child 1	26.5	160	0	0	0	219	0	0	885	0	0%	54.75	203.5	22.97%	1
MUd04	Parent	271.8	268.8	4.5	0	0	150	0	0	1594	21.75	1.35%	56.50	229.00	14.37%	
MUd04S	Child 1	21	142.5	27.75	0	0	0	0	0	709.5	37	5.21%	0	0	0%	0.5
MUd05	Parent	291	104	60.25	0	0	0	0	0	2500	294.75	11.79%	0	0	0%	
MUd05D	Child 1	228.25	64.75	15.5	0	0	4	0	0	1168	68.5	5.86%	1	5.25	0.45%	0.5
MUd06	Parent	207	86.75	0	0	27	0	0	0	1159	0	0%	6.75	32.00	2.76%	
MUd06S	Child 1	72.5	42.5	3	0	15	0	0	0	1028	15.5	1.51%	3.75	16.75	1.63%	0.5
MUd07	Parent	280.5	181.8	8.75	0	0	20	0	0	1558.75	46.50	2.98%	5	16.00	1.03%	
MUd07S	Child 1	282.8	100.3	0	0	0	0	0	0	1079.25	0	0%	0	0	0%	1.25
MU01	Parent	163.75	268.75	0	0	58	125	0	0	1865.5	0	0%	45.75	205.00	10.99%	
MU01S	Child 1	113.75	75	6.25	0	26	0	209	0	1277.5	31.5	2.47%	58.75	180.5	14.13%	1.25
MU02	Parent	50	167	5	0	12	352	125	0	2299	23.00	1%	122.25	411.50	17.90%	
MU02S1	Child 1	60.75	4	0	0	20	86	0	0	633.5	0	0%	26.5	118.8	18.75%	1
MU02S2	Child 2	67.5	19	0	0	34	50	0	0	966.75	0	0%	21	115	11.90%	1.5
MU03	Parent	198	200.3	0	0	20	304	0	0	2162.75	0	0%	81	323.12	14.94%	
MU03S	Child 1	331	190.8	0	0	0	120	0	0	1160.75	0	0%	30	125	10.77%	0.5
MU04	Parent	32	105.8	0	15	0	42	0	0	1164.75	0	0%	14.25	54.25	4.66%	
MU04S1	Child 1	33.5	48	26.5	10	68	185	0	43.75	1223	117.75	9.63%	65.75	263.5	21.55%	2.25
MU04S2	Child	90.75	41.25	2.75	10	40	202	0	0	992.5	10.5	1.06%	63	265.5	26.75%	1.75

	2															
MU05	Parent	95.75	175.3	28.5	0	72	113	0	0	1627.75	139.25	8.55%	46.25	206.75	12.70%	
MU05S	Child 1	160.5	50.5	18.25	10	0	118	0	0	1255.75	92.25	7.35%	32	145	11.55%	0.75
MU06	Parent	134.3	102.5	0	25.5	15	169	0	0	1317.75	0	0%	52.38	240.00	18.21%	
MU06D	Child 1	328.75	65	0	0	0	0	0	0	1249.5	0	0%	0	0	0%	0
Parent Group Mean Intakes										1699.81	50.25	2.55%	35.88	144.16	8.23%	
Child Group Mean Intakes										1036.33	27.98	2.50%	29.92	122.25	12.21%	

* Average number of HED snacks: eating more than one type of HED snacks at snack times was considered as one snack.
